# A case of a paediatric chondromyxoid fibroma-like osteosarcoma

**DOI:** 10.1093/bjrcr/uaaf011

**Published:** 2025-03-01

**Authors:** Khaoula Boumeriem, Iliass Bourekba, Nazik Allali, Latifa Chat, Siham El Haddad

**Affiliations:** Department of Radiology, Children’s Hospital of Rabat, Rabat MA 10000, Morocco; Department of Radiology, Children’s Hospital of Rabat, Rabat MA 10000, Morocco; Department of Radiology, Children’s Hospital of Rabat, Rabat MA 10000, Morocco; Department of Radiology, Children’s Hospital of Rabat, Rabat MA 10000, Morocco; Department of Radiology, Children’s Hospital of Rabat, Rabat MA 10000, Morocco

**Keywords:** chondromyxoid fibroma-like osteosarcoma, paediatric CMF-OS, CMF-OS

## Abstract

Chondromyxoid fibroma-like osteosarcoma (CMF-OS) is an exceptionally rare and low-grade variant of osteosarcoma, as classified by the World Health Organization. Misdiagnosis is common in CMF-OS, often leading to delays in definitive surgical intervention. CMF-OS exhibits variable imaging features, frequently mimicking chondromyxoid fibroma. It may present as osteolytic, osteogenic, or expansive lesions, often associated with soft tissue invasion, cortical disruption, and occasionally a periosteal reaction. Cases have been reported in diverse anatomical locations, including the craniofacial region and bones of the lower limbs. Histologically, CMF-OS is distinguished by its unique mucoid appearance, characterized by loose aggregates of stellate and spindle-shaped tumour cells embedded within a highly myxoid stroma. Surgical resection remains the cornerstone of treatment for CMF-OS, emphasizing the importance of accurate diagnosis to facilitate timely and appropriate management.

## Case report

**Figure 1.**
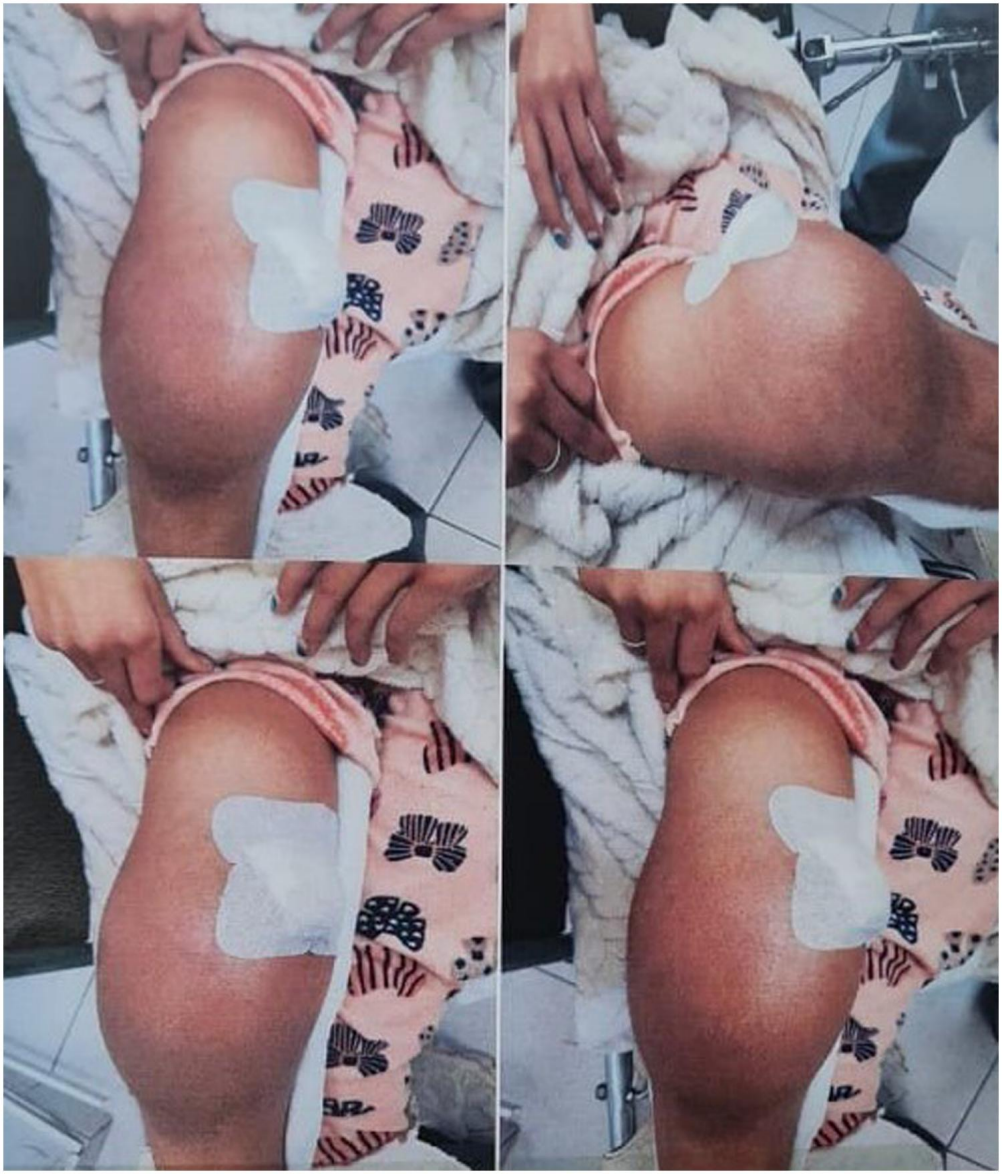
Clinical image of the patient showing an important swelling of the distal extremity of the right thigh.

A 12-year-old girl with no significant medical history presented to the paediatric surgery department with a painful swelling of the right thigh, resulting in a limp. There were no associated systemic symptoms, such as fever or weight loss. On physical examination, a prominent swelling was noted at the distal end of the right femur, accompanied by restricted mobility of the affected limb. No signs of local inflammation were observed (Figure 1).

**Figure 2.**
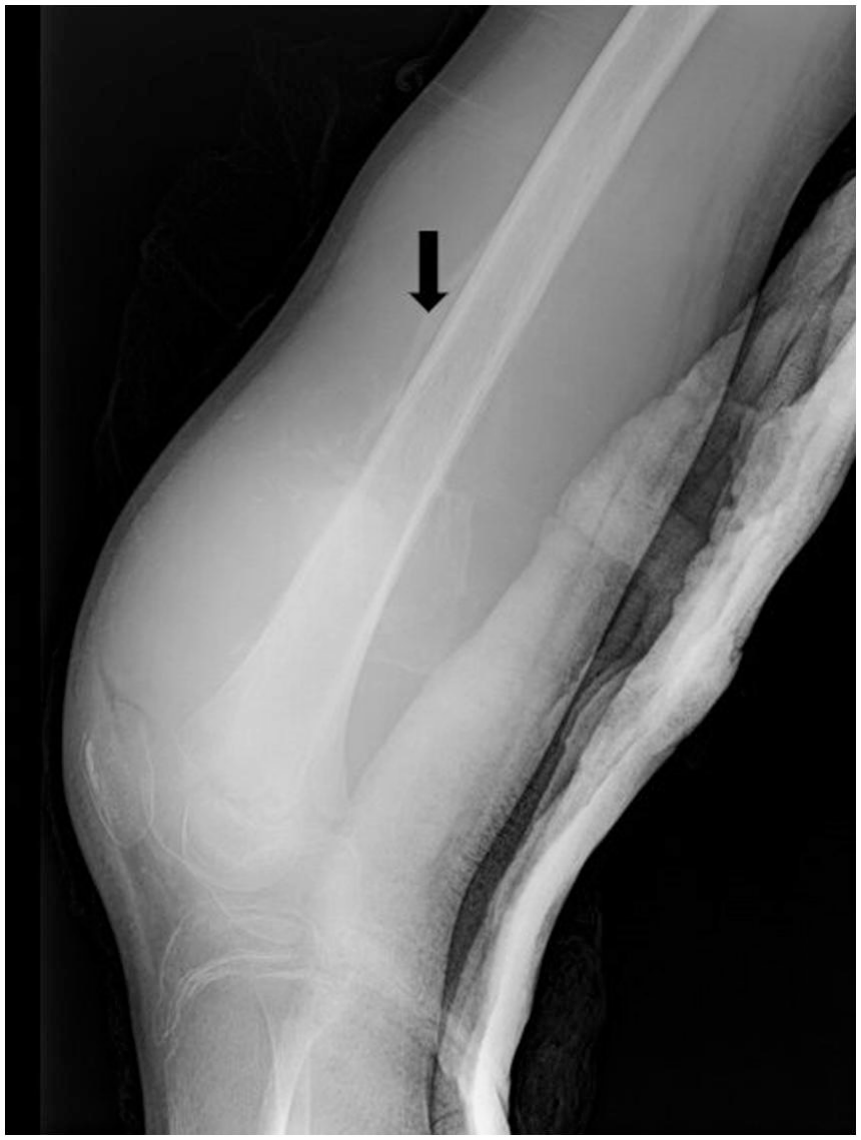
Plain radiography showing a diaphyseal–metaphyseal periosteal reaction (sunburst-like) of the inferior extremity of the right femur (black arrow).

Initial plain radiographs revealed a diaphyseal–metaphyseal periosteal reaction with a sunburst-like appearance at the distal end of the right femur (Figure 2).

Subsequent CT imaging, performed at an external facility with no available records, revealed a hypodense, septated cortico-medullary lesion extending from the metaphysis to the diaphysis of the distal third of the right femur, associated with medullary hyperdensity. The lesion exhibited cortical irregularities, periosteal reaction, circumferential peri-osseous extension, and displacement of the adjacent soft tissues.

**Figure 3.**
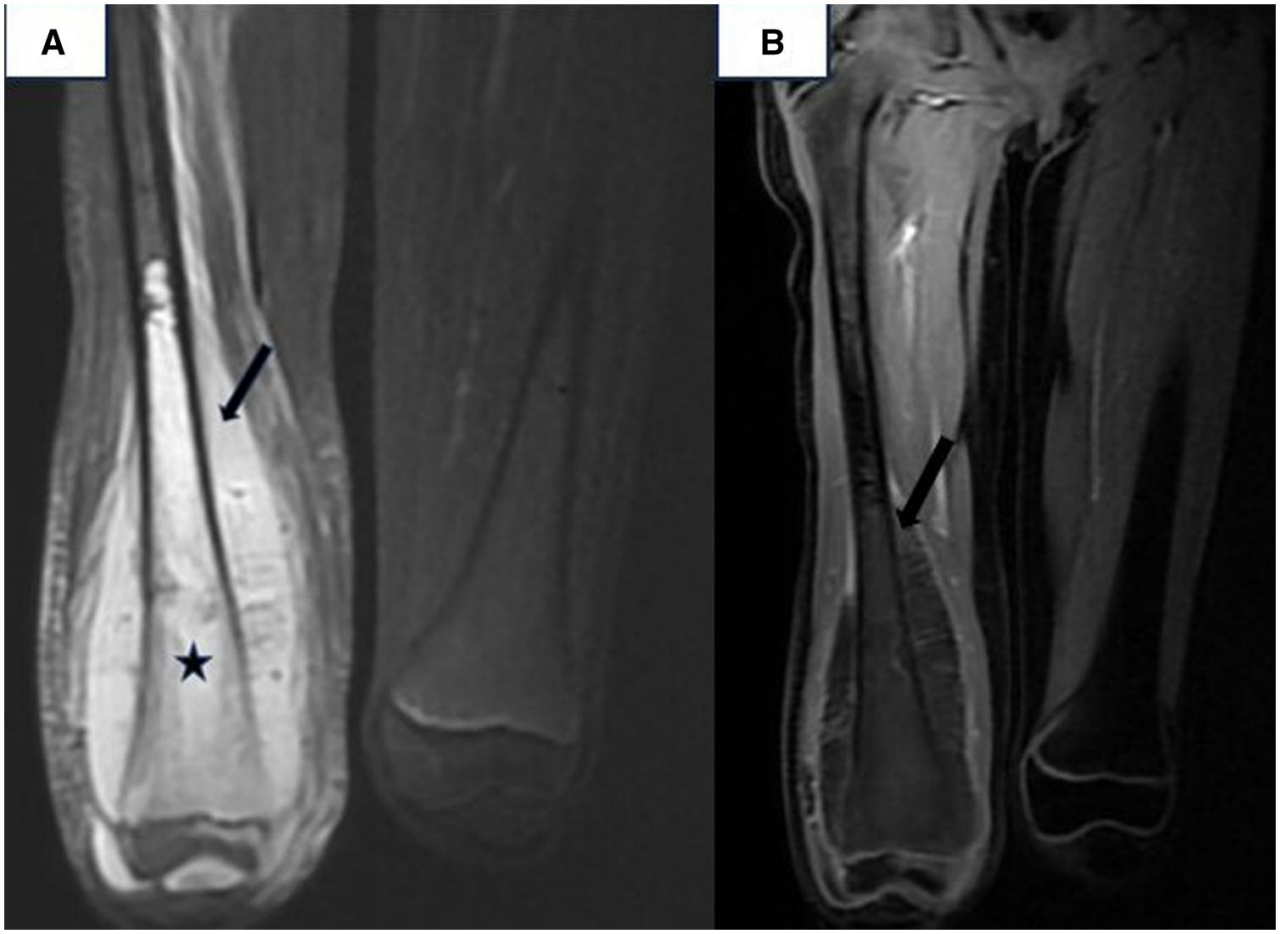
(A) MRI coronal T1 sequence after administration of gadolinum and MRI coronal FAT SAT sequence demonstrating a osteolytic lesion that involves the diaphysis, metaphysis, and epiphysis of the distal right femur (black star) with soft tissue invasion (black arrow).

**Figure 4.**
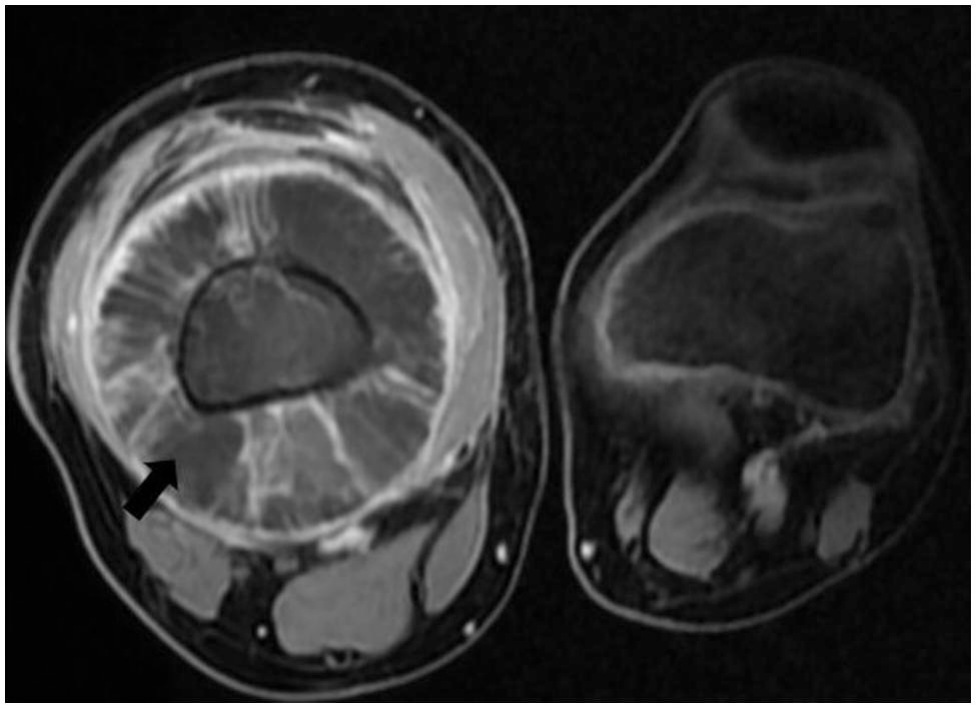
MRI axial sequence after administration of gadolinum demonstrating a osteolytic lesion that involves the diaphysis, metaphysis, and epiphysis of the distal right femur with heterogenous enhancement (black star).

MRI further characterized the lesion as an expansive, osteolytic tumour involving the metaphysis, diaphysis, and epiphysis of the femur. The tumour infiltrated both cortical and medullary bone and extended into adjacent soft tissues. On MRI, the lesion was isointense on T1-weighted images, hyperintense on T2-weighted sequences, and exhibited heterogeneous enhancement following gadolinium administration. It invaded the distal femoral epiphysis and was associated with intra-articular effusion in the knee joint. The tumour also compressed adjacent muscles, including the gracilis, sartorius, semitendinosus, and biceps femoris, as well as the vastus intermedius, vastus lateralis, and vastus medialis muscles, all of which appeared atrophic. The lesion displaced the popliteal vascular pedicle without compromising its patency (Figures 3 and 4).

**Figure 5.**
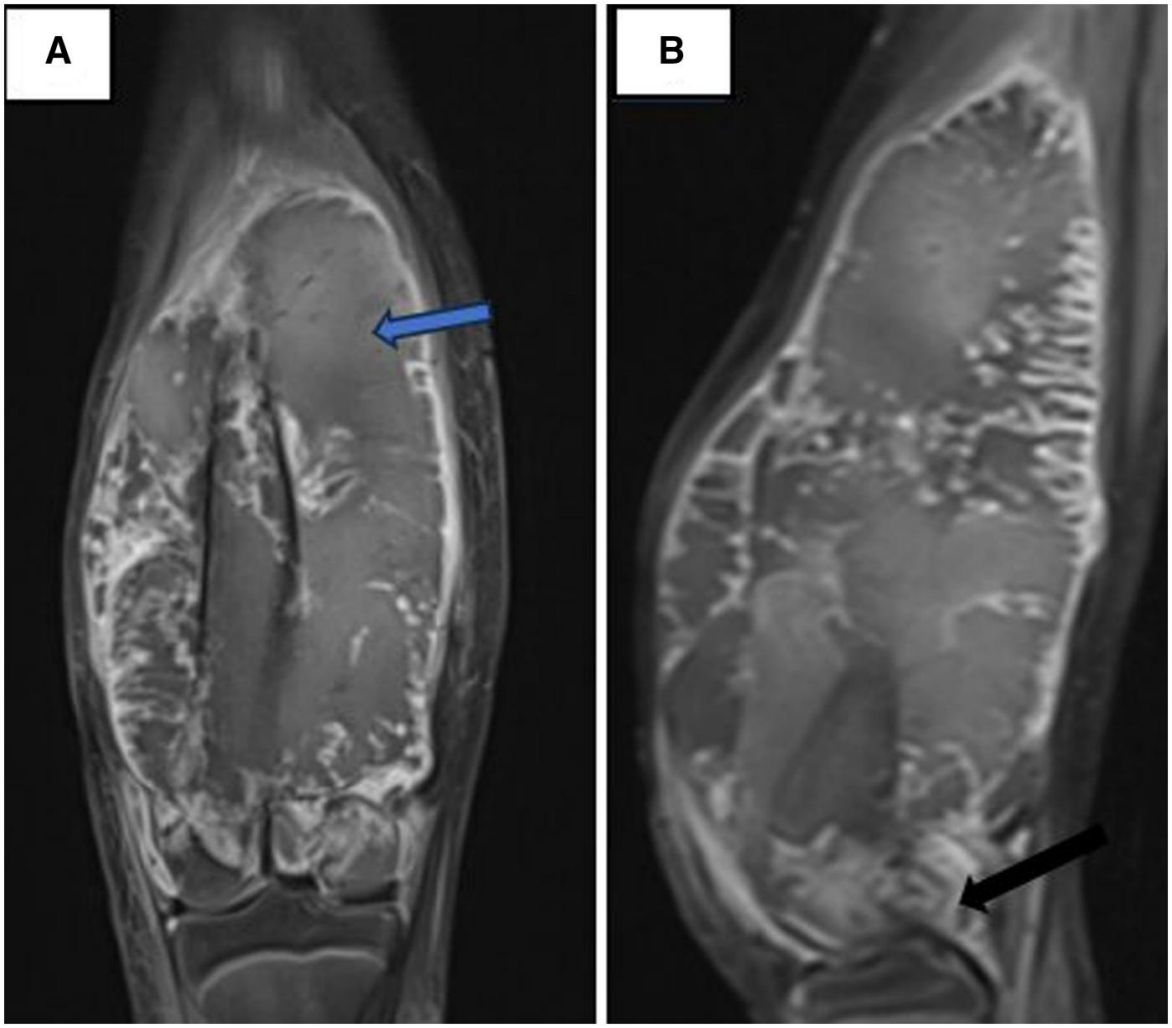
(A) MRI coronal T1 sequence after administration of gadolinium showing a lobulated heterogenous lesion with soft tissue invasion (blue arrow). (B) MRI sagittal T1 sequence after administration of gadolinium showing the extension of the mass to meniscal and ligamentous structures of the ipsilateral knee (black arrow).

Three months later, follow-up MRI revealed significant progression of the lesion, characterized by an aggressive pattern with lobulated margins. The lesion exhibited heterogeneous T1 hyperintensity, suggestive of intralesional haemorrhage, and invaded the femoral condyles and distal femoral physis. It extended to the meniscal and ligamentous structures of the knee (Figure 5).

A surgical biopsy was performed. Histopathological examination revealed a tumour composed of medium-sized stellate and spindle-shaped cells with hyperchromatic nuclei, arranged in a pseudolobulated architecture within a myxoid and cartilaginous stroma. These lobules were separated by fibrovascular septa containing multinucleated giant cells. Areas of higher cellular density with fibrous stroma, focal tumoural osteogenesis, and necrosis were also observed. Mitoses, including atypical figures, were frequent and estimated at 15 per 10 high-power fields. These findings supported the diagnosis of a chondromyxoid fibroma-like osteosarcoma (CMF-OS) (Figure S6).

Staging imaging revealed pulmonary nodules, raising suspicion of metastatic disease. The patient received one cycle of neoadjuvant chemotherapy and was scheduled for amputation. Unfortunately, she was subsequently lost to follow-up.

## Discussion

CMF-OS is an exceedingly rare subtype of osteosarcoma, classified as a low-grade variant by the World Health Organization (WHO). It can occur in both adults, typically during the third or fourth decade of life, and in the paediatric population. Misdiagnosis is common in both imaging and histopathological evaluations, complicating treatment management and increasing the risks of dedifferentiation, recurrence, and metastasis.^1^^,^^2^

The first case of CMF-OS was reported in 1989. It was initially described as one of the four variants of low-grade primary central (intramedullary) osteosarcoma. These variants, often resembling benign processes histologically, include osteoblastoma-like, fibrous dysplasia-like, non-ossifying fibroma-like, and chondromyxoid fibroma-like subtypes. The limited cases reported in the literature document various locations such as the craniofacial region, proximal tibia, distal femur, middle phalanx of the fifth finger, and iliac crest, with even fewer cases in the paediatric population.^2^

Clinical presentation varies depending on the tumour’s location. Most patients report a symptomatic mass, swelling, or pain. Unlike conventional osteosarcoma, elevated levels of alkaline phosphatase and lactate dehydrogenase are often absent in CMF-OS, limiting the utility of these biomarkers for diagnosis.^3^

On imaging, CMF-OS demonstrates variable radiographic and cross-sectional imaging appearances, often mimicking chondromyxoid fibroma. The lesion can present as osteolytic, osteogenic, or expansive, with or without calcifications. Most commonly, CMF-OS manifests as an expansive osteolytic lesion with incomplete trabeculation, cortical disruption, and soft tissue invasion, occasionally accompanied by a periosteal reaction. In contrast, chondromyxoid fibroma typically appears as a lobulated or oval, eccentric lytic lesion with well-defined sclerotic margins and geographic bone destruction, sometimes with internal septations.^1^^,^^3^

Histopathological wise: Macroscopically, CMF-OS exhibits a characteristic glistening mucoid appearance. Microscopically, the tumour comprises aggregates of spindle or stellate cells within a highly myxoid stroma, separated by fibrovascular septa, closely resembling chondromyxoid fibroma. Additional features, such as nuclear atypia and a high Ki-67 proliferation index, help differentiate CMF-OS from benign lesions. Differential diagnoses include chondromyxoid fibroma, chondrosarcoma, and other low-grade central osteosarcomas.^2^^,^^4^

Surgical resection remains the cornerstone of treatment for CMF-OS. Due to its relatively invasive biological behaviour and limited response to chemotherapy or radiotherapy, CMF-OS carries a high risk of recurrence and metastasis, underscoring the need for timely and accurate diagnosis.^4^

## Learning points

- Chondromyxoid fibroma-like osteosarcoma (CMF-OS) is an exceptionally rare and low-grade subtype of osteosarcoma, classified by the World Health Organization (WHO).
- Misdiagnosis is common, leading to delays in surgical intervention.
- CMF-OS exhibits variable imaging features, often mimicking chondromyxoid fibroma, with presentations as osteolytic, osteogenic, or expansive lesions. Cortical disruption, soft tissue invasion, and periosteal reactions are frequently observed.
- Cases have been reported in diverse anatomical sites, including the craniofacial region and lower limb bones.
- Histologically, CMF-OS is characterized by a mucoid appearance with loose cell aggregates embedded in a highly myxoid stroma.
- Surgical resection is the primary treatment for CMF-OS due to its invasive nature and potential for recurrence and metastasis.

## Informed consent

Written informed consent was obtained from the patient for publication of this case review, including accompanying images.

## Supplementary Material

uaaf011_Supplementary_Data
