# Supplementary material for: A case of a paediatric chondromyxoid fibroma-like osteosarcoma
Source: BJR Case Rep. 2025 Mar 1;11(2):uaaf011. doi: 10.1093/bjrcr/uaaf011 (PMC11922549; doi:10.1093/bjrcr/uaaf011)
Supplement: uaaf011_Supplementary_Data [file uaaf011_supplementary_data.zip › Supplementary caption.docx]

**Figure 6:** Histological examination compatible with a chondromyxoid fibroma like osteosarcoma
